# Frequency of anterolateral ligament tears and ramp lesions in patients with anterior cruciate ligament tears and associated injuries indicative for these lesions—a retrospective MRI analysis

**DOI:** 10.1007/s00330-023-09444-z

**Published:** 2023-02-18

**Authors:** Nikolaus Stranger, Christian Kaulfersch, Georg Mattiassich, Jürgen Mandl, Peter A. Hausbrandt, Dieter Szolar, Helmut Schöllnast, Manfred Tillich

**Affiliations:** 1 Division of General Radiology, Department of Radiology, Medical University of Graz, Graz, Austria; 2 Department of Trauma Surgery, Klinik Diakonissen Schladming, Schladming, Austria; 3 Trauma Hospital Graz, Graz, Austria; 4 Diagnostikum Graz, Graz, Austria; 5 Institute of Radiology, LKH Graz II, Göstinger Strasse 22, 8020 Graz, Austria

**Keywords:** Knee joint, Magnetic resonance imaging, Anterior cruciate ligament tears, Anterolateral ligament, Ramp lesion

## Abstract

**Objectives:**

To assess the frequency of anterolateral ligament (ALL) tears and ramp lesions (RL) detected with MRI in patients with anterior cruciate ligament (ACL) tears and to describe associated injuries indicative for these lesions.

**Methods:**

In this retrospective study, 164 patients with surgically verified ACL tears were included. Preoperative MRI scans were reviewed for ALL tears and different types of RL. All coexisting meniscal tears, tears of the medial (MCL) and lateral collateral band (LCL), and posterior-medial tibial bone marrow edema (BME) were recorded. The frequency of ALL tears and RL was assessed and coexisting injuries were correlated using Pearson’s chi-square test. A *p* < 0.05 was defined as statistically significant. In cases of multiple testing, Bonferroni’s correction was applied.

**Results:**

ALL tears and RL combined were detected in 28 patients (17.1%), ALL tears in 48 patients (29.3%), and RL in 54 patients (32.9%) which were significantly associated to each other. ALL tears were significantly associated with tears of the posterior horn of the lateral meniscus (PHLM), BME, and with tears of the LCL and MCL. RL were significantly associated with tears of the posterior horn of the medial (PHMM) and PHLM, with BME, and with tears of the LCL.

**Conclusions:**

ACL tears are associated with RL or ALL tears in about one-third of cases and with both lesions combined in about one-fifth of cases. ALL tears and RL are significantly associated with additional posttraumatic injuries, which can thus be indicative of these lesions.

**Key Points:**

*• ACL tears were associated with ramp lesions or ALL tears in about one-third of the cases.*

*• Ramp lesions and ALL tears were significantly associated with each other, tear in the PHLM, tear in the LCL, and BME.*

*• ALL tears were more frequently associated with instable classified ramp lesion type 4b and type 5.*

**Supplementary Information:**

The online version contains supplementary material available at 10.1007/s00330-023-09444-z.

## Introduction

Despite increasing awareness of anterolateral ligament (ALL) tears and ramp lesions in patients with anterior crucial ligament (ACL) tears, these lesions are nevertheless significantly underdiagnosed due to lack of preoperative diagnosis on MRI or inadequate visualization of the PHMM during arthroscopy [1]. In addition, clinical assessment of ALL tears is difficult [2]. The ALL inhibits internal rotation of the tibia and acts as a stabilizer against internal rotation when the knee joint is flexed [3]. Tears of the ALL result in an increased anterior tibial translation and increased internal rotation [4]. Ramp lesions result in significantly increased anterior translational mobility and external rotational instability of the tibia [5]. In the clinical examination, all injury and ramp lesions demonstrated an increase in motion in the pivot-shift test [6–8]. In patients with ACL tear and ALL tear or ramp lesion, stability of the knee joint is often not fully restored with ACL reconstruction alone, but it can be restored by additional reconstruction of the ALL or treatment of the ramp lesion [5–9]. Because untreated ramp lesions can lead to permanent instability in the knee joint due to destabilization of the PHMM; this results in a higher ACLR failure rate, increased cartilage wear, and degeneration [6, 10]. Stability in the pivot-shift test can only be restored after ACL reconstruction and operative treatment of the ALL tear or ramp lesion [6–11]. Furthermore, Sonnery-Cottet et al. [12] showed that ALL reconstruction reduced ACL reconstruction rerupture rates by at least 2.5-fold.

Therefore, preoperative assessment of ALL tear and ramp lesion in patients with ACL tear is an important guide for surgeons in selecting appropriate techniques for knee stabilization. Inconsistent frequency of ALL tears detected with MRI in patients with ACL tears has been reported that reached from 26 to 62% [13, 14]. The frequency of ramp lesions detected with arthroscopy was 15.5% in a study on 2156 patients with ACL tears, whereas 68.4% of these lesions were described in the preoperative MRI report [15]. Limited literature is available on the frequency of ALL tears and different types of ramp lesions in a single cohort of patients with ACL tears, whereas both lesions may lead to persistent instability after ACL reconstruction if untreated. In addition, limited literature is available on associated injuries, which could be indicative of a ramp lesion and an ALL tear in MRI. Therefore, the aim of our study was to assess the frequency of ALL tears and different types of ramp lesions in MRI of patients with verified ACL tears as well as to analyze associated injuries to these lesions.

## Materials and methods

### Patient selection

The ethics committee of the Medical University of Graz approved this study and informed consent was waived due to the retrospective study design.

A computer-based data search of medical records yielded 164 consecutive patients (mean age ± SD, 34.6 ± 13.2 years; range, 9–62 years; female, 49.5%; male, 50.5%). All patients with arthroscopic confirmation of an acute or chronic ACL tear between January 2017 and March 2020, who had a preoperative MRI of the involved knee available and also underwent surgical reconstruction of the affected knee, were included in this study. An ACL tear was considered acute if the MRI was performed within 6 months of the injury and was considered chronic if the MRI was performed more than 6 months after the injury. We did not include patients without a complete ACL tear, patients without a preoperative MRI, or patients without ACL tear. We categorized the patients after age into three groups (9–20, 21–40, 41–62). The mean time interval (± SD) between MRI examination and ACL reconstruction was 44.4 ± 78.9 days (range, 0–560 days). We did not compare the findings identified on MRI compared to arthroscopy due to the retrospective aspect of the study, the missing classification of ramp lesions after Greif et al. [16] by the time of the arthroscopy, and the missing visibility of the all in the arthroscopy.

### MR imaging protocol and interpretation

All MRI scans were performed either on a 1.5 T scanner (Magnetom Symphony TIM, Siemens) or a 3 T scanner (Magnetom Skyra, Siemens) using dedicated knee coils. For both scanners, the protocol included proton-density (PD) weighted fat-suppressed images in the coronal, sagittal, and transverse planes as well as T1 weighted images in the coronal plane. Detailed protocols for both scanners are provided in Supplemental Material 1.

Two radiologists with 15 years of experience in musculoskeletal imaging reviewed all MRI scans in consensus. At the time of the review, the radiologist was informed that the patient had an arthroscopically confirmed diagnosis of an ACL tear, but did not have access to the initial MRI reports, operation reports, or clinical information. Ramp lesions were defined according to Greif et al. [16] as longitudinal tears extending in the mediolateral direction located in the PHMM or the meniscotibial or meniscocapsular junction including also vertical tears perpendicular to the tibial plateau and tears oblique to the tibial plateau. In addition, the following classifications of ramp lesion were used according to Greif et al. [16]: type 1—tears of the meniscocapsular ligament; type 2—superior partial tears of the PHMM with intact meniscocapsular junction; type 3A—vertical peripheral tears of the inferior border of the PHMM with an intact meniscotibial ligament; type 3B—tears of the meniscotibial ligament, or tears of the insertion at the medial meniscus; type 4A—longitudinal, vertical complete tears of the red-red zone of the PHMM with intact meniscotibial and meniscocapsular attachments to the torn meniscal fragment; type 4B—complete tear of the meniscocapsular and meniscotibial attachments; type 5—two separately running tears in the red-red zone of the PHMM. Type 1 and type 2 lesions were classified as stable lesions; types 3 to 5 lesions were classified as unstable lesions [16]. ALL tears were defined as partial or complete discontinuity or bony avulsion of the ALL. Periligamentous edema with continuous low signal intensity fibers was not considered as ALL tears. Complete tears of the MCL or LCL, tears of the medial or lateral meniscus, and posterior-medial tibial bone marrow edema were recorded as potentially associated injuries.

### Statistical analysis

Data are provided as absolute numbers and frequencies. The correlation between ALL tears and ramp lesions was tested using Pearson’s chi-square test. A *p* < 0.05 was defined as statistically significant. The significance of the difference in stability of ramp lesions between patients with and without ALL tears was tested using Fisher’s exact test, whereby a *p* < 0.05 was defined as statistically significant. The correlation of ALL tears and ramp lesions with coexisting injuries was tested for statistical significance using Pearson’s chi-square test and Fisher’s exact test. After Bonferroni’s correction of multiple testing, a *p* < 0.007 was defined as statistically significant. Statistical analysis was performed with IBM SPSS Statistics (Version 27, IBM).

## Results

**Fig. 1:**
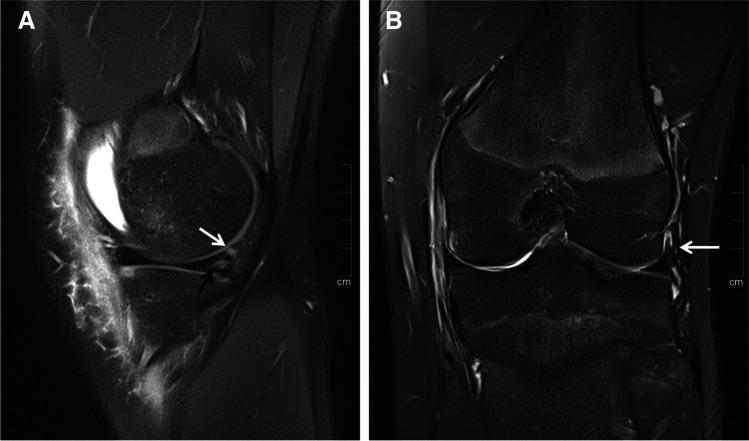
19-year-old male with acute ACL tear (not shown). **A** Sagittal, proton-weighted, fat-suppressed TSE sequence shows a type 1 ramp lesion with a peripheral, vertical tear of the meniscocapsular ligament (white arrow). In the PHMM, an associated intrasubstance tear is shown (black arrow). **B** Coronal, proton-weighted, fat-suppressed TSE sequence shows a normal ALL without signs of an injury (arrow)

The frequencies of ALL tears, ramp lesions, and combined ALL tears and ramp lesions as well as the frequencies of potentially associated injuries are provided in Table 1. Among the 54 patients (32.9%) with ramp lesions, seven ramp lesions (13%) were classified as stable lesions (Fig. 1) and 47 ramp lesions (87%) were classified as unstable lesions. Table 2 provides details on ramp lesion characterization.

**Table 1:** Frequencies of ALL tears, RL, ALL tears, and RL combined as well as the frequencies of potentially associated injuries

Lesions/tears	Number of patients (*n* = 164)	Prevalence
ALL	48	29.3%
RL	54	32.9%
ALL + RL	28	17.1%
BME	34	20.7%
MCL	39	23.8%
LCL	11	6.7%
AHMM	8	4.9%
PHMM	57	34.8%
AHLM	9	5.5%
PHLM	48	29.3%

*ALL* anterolateral ligament, *RL* ramp lesion, *BME* bone marrow edema in the posterior-medial tibia, *MCL* medial collateral ligament, *LCL* lateral collateral ligament, *AHMM* anterior horn of medial meniscus, *PHMM* posterior horn of medial meniscus, *AHLM* anterior horn of lateral meniscus, *PHLM* posterior horn of lateral meniscus

**Table 2:** RL classification according to Greif et al

RL type	Stability	Number of patients	Prevalence
1	Stable	6	11.1%
2	Stable	1	1.9%
3A	Unstable	5	9.3%
3B	Unstable	12	22.2%
4A	Unstable	16	29.6%
4B	Unstable	11	20.4%
5	Unstable	3	5.6%

**Fig. 2:**
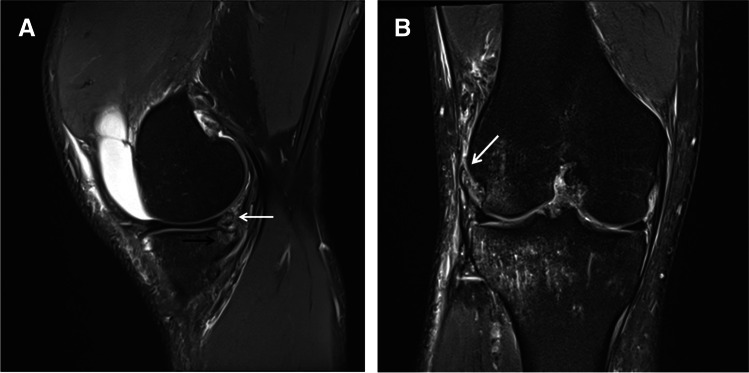
37-year-old male with acute ACL tear (not shown). **A** Sagittal, proton-weighted, fat-suppressed TSE sequence shows a type 5 ramp lesion with double vertical longitudinal tear of the peripheral red-red zone of the PHMM (white arrow) with meniscotibial- and meniscocapsular-junctional separation. Note the subchondral BME adjacent to the type 5 ramp lesion (black arrow). **B** Coronal, proton-weighted, fat-suppressed TSE sequence shows a complete tear of the proximal part of the ALL with associated periligamentary edema (arrow)

**Fig. 3:**
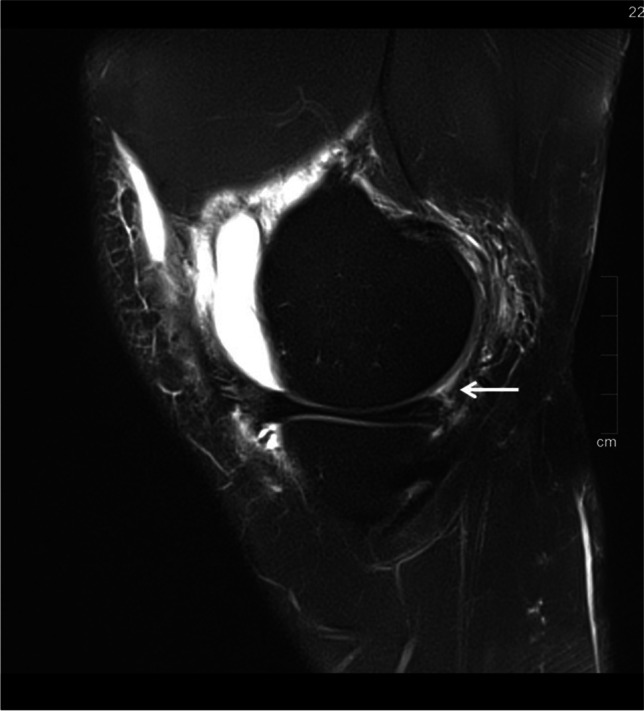
17-year-old male with acute ACL tear (not shown). Sagittal, proton-weighted, fat-suppressed TSE sequence shows a type 2 ramp lesion with superior partial tear of the PHMM with intact meniscocapsular junction (white arrow)

**Fig. 4:**
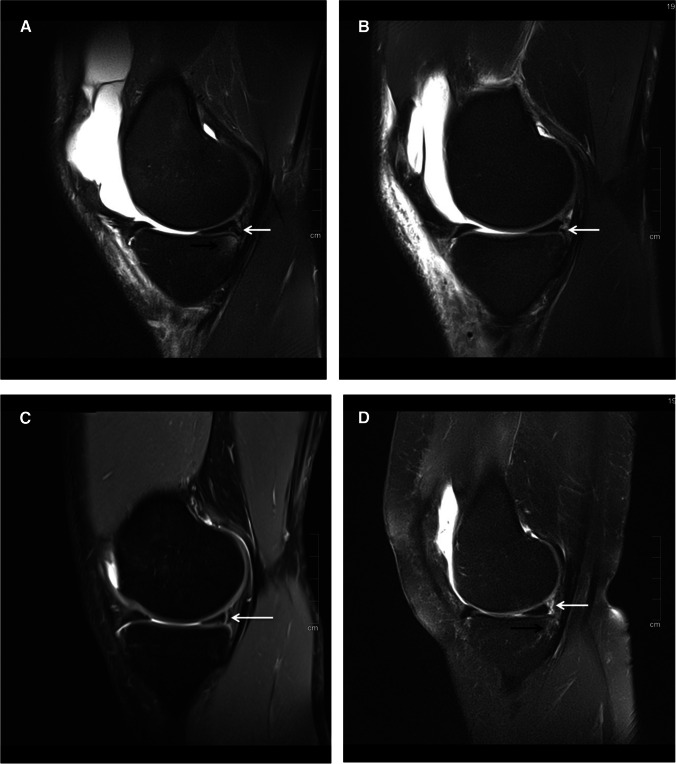
**A** 26-year-old male with acute ACL tear (not shown). Sagittal, proton-weighted, fat-suppressed TSE sequence shows a type 3A ramp lesion with vertical peripheral tear of the inferior border of the PHMM with an intact meniscotibial ligament (white arrow). Note the subchondral BME adjacent to the type 3A ramp lesion (black arrow). **B** 30-year-old male with acute ACL tear (not shown). Sagittal, proton-weighted, fat-suppressed TSE sequence shows a type 3B ramp lesion with tear of the insertion of the meniscotibial attachment at the medial meniscus (white arrow). **C** 42-year-old male with acute ACL tear (not shown). Sagittal, proton-weighted, fat-suppressed TSE sequence shows a type 4A ramp lesion with longitudinal, vertical complete tear of the red-red zone of the PHMM with intact meniscotibial and meniscocapsular attachments to the torn meniscal fragment (white arrow). **D** 21-year-old male with acute ACL tear (not shown). Sagittal, proton-weighted, fat-suppressed TSE sequence shows a type 4B ramp lesion with complete tear of the meniscocapsular and meniscotibial attachments (white arrow). Note the subchondral BME adjacent to the type 4B ramp lesion (black arrow)

ALL tears and ramp lesions were significantly correlated to each other (*p* < 0.001). Out of 48 patients with ALL tears, 28 patients (58.3%) showed combined ramp lesions, whereas 20 patients (41.7%) did not. Out of 54 patients with ramp lesions, 28 patients (51.9%) showed combined ALL tears, whereas 26 patients (48.1%) did not. Patients with ALL tears showed different types of ramp lesions compared to patients without ALL tears. Type 4b and type 5 ramp lesions were nearly exclusively seen in patients with ALL tears (Fig. 2), whereas patients without ALL tears type 3b and type 4a lesions were the most frequent ones (Table 3). There was no statistically significant difference in the stability of ramp lesions between patients with and without ALL tears. In patients with ALL tears and ramp lesions combined, 25/28 patients (89.3%) showed unstable ramp lesions and 3/28 patients (10.7%) showed stable ramp lesions (Fig. 3), whereas in patients without ALL tears, 22/26 patients (84.6%) showed unstable ramp lesions (Fig. 4A–D) and 4/26 (15.4%) patients showed stable ramp lesions (*p* > 0.05).

**Table 3:** Correlation between ALL tears and different types of ramp lesions (*p* = .015)

ALL tear	Ramp lesion type						
	1	2	3a	3b	4a	4b	5
No	3 (11.5%)	1 (3.8%)	3 (11.5%)	7 (26.9%)	9 (34.6%)	3 (11.5%)	0
Yes	3 (10.7%)	0	2 (7.1%)	5 (17.9%)	7 (25%)	8 (28.6%)	3 (10.7%)

BME in the posterior-medial tibia, PHLM tears, and MCL tears were statistically significantly associated with ALL tears; LCL tears were borderline significant (*p* < 0.001–0.003, Table 4). BME in the posterior-medial tibia, LCL tears, and PHLM and PHMM tears were statistically significantly associated with ramp lesions (*p* < 0.001–0.006, Table 5).

**Table 4:** Correlation between ALL tears and potentially accompanying injuries (*BME* bone marrow edema in the posterior-medial tibia, *MCL* medial collateral ligament, *LCL* lateral collateral ligament, *AHMM* anterior horn of medial meniscus, *PHMM* posterior horn of medial meniscus, *AHLM* anterior horn of lateral meniscus, *PHLM* posterior horn of lateral meniscus)

	Patients with ALL injury		Patients without ALL injury		
Accompanying injuries	Number of patients	Prevalence	Number of patients	Prevalence	*p* value^#^
BME	20	41.7%	14	12.1%	< .007**
MCL	19	39.6%	20	17.2%	< .007*
LCL	7	14.6%	4	3.4%	.009*
AHMM	3	6.3%	5	4.3%	.600
PHMM	19	39.6%	38	32.8%	.404
AHLM	4	7.1%	5	4.6%	.894
PHLM	27	56.3%	21	18.1%	< .007**

^#^After Bonferroni-Holm’s correction

^*^p < .05, **p < .01, ***p < .001

**Table 5:** Correlation between RL and potentially accompanying injuries (*BME* bone marrow edema in the posterior-medial tibia, *MCL* medial collateral ligament, *LCL* lateral collateral ligament, *AHMM* anterior horn of medial meniscus, *PHMM* posterior horn of medial meniscus, *AHLM* anterior horn of lateral meniscus, *PHLM* posterior horn of lateral meniscus)

Accompanying injuries	Patients with RL		Patients without RL		*p* value^#^
	Number of patients	Proportion	Number of patients	Proportion	
BME	34	63%	0	0.0%	< .007**
MCL	16	29.6%	23	20.9%	.218
LCL	8	14.8%	3	2.7%	< .007**
AHMM	1	1.9%	7	6.4%	.274
PHMM	35	64.8%	22	20%	< .007**
AHLM	4	7.4%	6	5.5%	.731
PHLM	24	44.4%	24	21.8%	< .007**

^#^After Bonferroni-Holm’s correction

^*^p < .05, **p < .01, ***p < .001

The prevalence of ramp lesions increases with age. Higher age and ramp lesions. were statistically significantly associated (*p* < 0.05), with 40.7% of ramp lesions occurring in the age group from 41 to 62.

ALL injuries showed no statistically significant correlation to an age group.

We found almost perfect interobserver agreement in the detection of ramp lesions (kappa = 0.99), both observers agreed on 53/54 ramp lesions (98.1%) and disagreed in 1 patient (1.9%). And almost perfect interobserver agreement in the detection of ALL injuries (kappa = 0.83, both observers agreed on 47/48 ALL injuries (97.9%) and disagreed in 1 patient (2.08%). We found substantial interobserver agreement in the classification of ramp lesions (kappa = 0.8).

## Discussion

In our study on MRI of patients with surgically proven ACL tears, ALL tears were observed in 29.3% and ramp lesions were observed in 32.9%, whereas ALL tears and ramp lesions combined occurred in 17.1%. The frequency of ALL tears and ramp lesions in our study is in accordance with the literature. Published prevalence rates for ALL tears observed in MRI of patients with ACL tears are highly variable due to different definitions of ALL tears. Claes et al. [17] reported ALL tears in 78.8% of patients with ACL tears, whereby in addition to complete or partial interruptions of the ALL continuity, irregularities of the continuity as well as perligamentous edema were considered tears. Kosy et al. [18] demonstrated ALL tears in only 10.7% of patients with ACL tears. In their study [18], only complete or partial interruptions of the ALL continuity and bony avulsions of ALL were scored as ALL tears as we did in our study. Gaunder et al. [13] reported a frequency of ALL tears in ACL-deficient knees of 28.2% and 39.2%, respectively, depending on the observer. Ramp lesions have been reported to occur in 9 to 42% of patients undergoing ACL reconstruction [1–19]. In an arthroscopy study on 2156 patients with ACL reconstruction, Thaunat et al. [15] reported a prevalence of ramp lesions of 15.5%. In that study, ramp lesions were classified according to a previous work of the authors [20], whereas this classification is comparable with the classification by Greif et al. [16] disregarding the subgroups in types 3 and 4 lesions. In that study, type 1 ramp lesions occurred in 47.9%, type 2 lesions in 4.8%, type 3 lesions in 11.4%, type 4 lesions in 28.7%, and type 5 lesions in 7.2% [15]. The authors reported that 68.4% of ramp lesions were detected with MRI and 31.6% were missed, type 3 lesions were most likely to be missed on MRI, while type 4 lesions were most likely to be detected [15]. This is only partly consistent with the present study as type 4 lesions were the most frequent lesions in our cohort (50%), but type 3 lesions were the second most frequent one (31.5%). In contrary, type 1 lesions, which were the most frequent lesions in the study by Thaunat et al. [15], were observed in only 11.1% in our cohort. The differences may be explained by interobserver reliability in ramp lesion classification that has been shown to be moderate at 0.56 [21]. We agree with that statement because we had only substantial agreement in interobserver reliability in ramp lesion classification, with the most variety in the differentiation between ramp lesion type 3a and type 3B.

In this study, ALL tears and ramp lesions were significantly correlated to each other, whereby ramp lesion type 4b was most frequently associated with ALL tears (28.6%) followed by type 4a lesions (25%). On the contrary, in patients without ALL tears, lower-type ramp lesions were detected. The currently available literature does not yet include studies that addressed the correlation between ALL injury and different ramp lesion types.

According to the literature, ramp lesions occur in significant association with a complete ACL tear, BME in the posterior-medial tibia, tear of the LM, ALL injury, and LCL tear [11, 22, 23]; this is in accordance with our results. In our study, ramp lesions were statistically significantly associated with BME in the posterior-medial tibia, LCL tears, ALL injuries, PHLM tears, and PHMM tears. ALL tears were significantly correlated with BME in the posteromedial tibia, ramp lesions, PHLM tears, and MCL tears, and were borderline significantly correlated with LCL tears. This is backed up by Van Dyck et al. [24] who demonstrated a significant association between ALL injuries, LCL tears, and BME in the posteromedial tibia in patients with ACL tears and showed a borderline significant correlation between ALL injuries and MCL tears in ACL-deficient knees [24]. And Song et al. [25] found a significant correlation between ALL injuries and MCL tears.

There were several limitations in this retrospective study. The first limitation of this study was that only ACL and PCL tears were confirmed by arthroscopy. Second, we did not differentiate between acute or chronic ACL tear, nor did we differentiate between proximal and distal ALL injury. The third limitation was that we did not compare 1.5-T and 3.0-T MR images to assess ramp lesion and ALL injury visibility. Hatayama et al. [26] found a higher sensitivity at 3-T than at 1.5-T for diagnosing ramp lesions, but the difference was not statistically significant. Taneja et al. [27] found comparable ALL detection rates at both field strengths. The fourth limitation was the small sample size of the study, especially for the individual subtypes of ramp lesions classified according to Greif et al. [16]. Due to the low number of these different types of ramp lesions, there was only limited information about concomitant injuries and their distribution.

In our study, ramp lesions and ALL injuries were both significantly associated with BME in the posteromedial tibia, tear of the PHLM, and tear of the LCL. Ninety-three percent of ramp lesions, associated with ALL injuries, were classified as unstable according to Greif et al. [16]’s classification. Type 4B ramp lesions were most commonly associated with ALL injuries. Subchondral BME was predominantly associated with unstable classified ramp lesion types 3–5. Knees with an ACL tear, tear of the PHLM, LCL tear, and BME in the posteromedial tibia may indicate a concomitant ramp lesion or ALL injury and can therefore be considered additional diagnostic signs in MRI.

## Supplementary Information

Below is the link to the electronic supplementary material.

Supplementary file1 (DOCX 22 KB)

## Abbreviations

ACL: Anterior cruciate ligament
AHLM: Anterior horn of the lateral meniscus
AHMM: Anterior horn of the medial meniscus
ALL: Anterolateral ligament
BME: Bone marrow edema in the posteromedial tibial plateau
LCL: Lateral collateral ligament
MCL: Medial collateral ligament
PCL: Posterior cruciate ligament
PHLM: Posterior horn of the lateral meniscus
PHMM: Posterior horn of the medial meniscus
RL: Ramp lesion, ramp lesions

## Declarations

### Guarantor

The scientific guarantor of this publication is Manfred Tillich.

### Statistics and biometry

Gerold Schwantzer and Franz Quehenberger, Medical Informatics, Statistics and Documentation, Medical University of Graz, Austria, kindly provided statistical advice for this manuscript.

### Study subjects or cohorts overlap

Study subjects or cohorts have been previously reported in a thesis in German language.

### Methodology

• retrospective

• diagnostic or prognostic study

• multicenter study
