# Supplementary material for: Frequency of anterolateral ligament tears and ramp lesions in patients with anterior cruciate ligament tears and associated injuries indicative for these lesions—a retrospective MRI analysis
Source: Eur Radiol. 2023 Feb 18;33(7):4833–41. doi: 10.1007/s00330-023-09444-z (PMC10290041; doi:10.1007/s00330-023-09444-z)
Supplement: Supplementary file 1 — Supplementary file1 (DOCX 22 KB) [file 330_2023_9444_MOESM1_ESM.docx]

Supplemental Material 1

Symphony TIM 1.5 T protocol (Siemens)

Proton-weighted fat-suppressed images in the coronal, sagittal and transverse axis were taken with the following settings: base resolution: 320/90, TR 3410ms, TE 60ms, slice thickness 3mm, FOV 160mm. Sagittal proton and T2 weighted TSE images were taken with the following settings: base resolution 384/70, TR 4090ms, TE 166/13ms, slice thickness 4mm, FOV 160mm. Coronal T1-weighted SE images were taken with the following settings: base resolution 320/90, TR 631ms, TE 14ms, slice thickness 3mm, FOV: 160mm.

Magnetom Skyra 3.0 T protocol (Siemens)

Coronal proton weighted fat suppressed images were taken with the following settings: base resolution 384/307, TR 3200ms, TE 25ms, slice thickness 3mm, FOV 100mm. Axial proton weighted fat suppressed images were taken with the following settings: base resolution 320/256, TR 4110ms, TE 35ms, slice thickness 3mm, FOV:100mm. Sagittal Dixon sequence images were taken with the following settings: base resolution 384/269, TR 2700ms, TE 33ms, slice thickness 3mm, FOV 100mm. Coronal T1-weighted images were taken with the following settings: base resolution 384/307, TR 582ms, TE 10ms, slice thickness 3mm, FOV: 100mm.
